# The impact of the 2019 revision of China’s drug administration law on the innovative output of pharmaceutical and biotechnology enterprises—a quasi-natural experimental study using sharp regression discontinuity design

**DOI:** 10.3389/fmed.2026.1592775

**Published:** 2026-04-01

**Authors:** Yanan Zhu, Yongfei Liu, Yuwen Chen, Lingquan Meng

**Affiliations:** 1 School of Business Administration, Shenyang Pharmaceutical University, Shenyang, China; 2 Drug Regulatory Research Base of NMPA, Research Institute of Drug Regulatory Science, Shenyang Pharmaceutical University, Shenyang, China

**Keywords:** China drug administration law, innovation capability, pharmaceutical and biological enterprises, policy evaluation, sharp regression discontinuity design

## Abstract

**Introduction:**

The “Drug Administration Law of the People’s Republic of China” has a profound impact on the innovation of pharmaceutical companies.

**Methods:**

This study analyzes Chinese pharmaceutical and biotechnology companies from 2014 to 2023 and employs a sharp regression discontinuity design (RDD) based on the 2019 revision of the Drug Administration Law to examine changes in innovation output before and after the policy, while exploring impact mechanisms from external conditions (financing constraints) and internal factors (human capital structure), with further heterogeneity analysis by firm size, region, and ownership.

**Results:**

The results show that the implementation of the 2019 revised Drug Administration Law significantly increased the number of patent applications by pharmaceutical and biotechnology enterprises, thereby promoting innovation output. Further analysis finds that the policy promotes corporate innovation through two pathways: easing financing constraints and facilitating adjustments in human capital structure.

**Discussion:**

This study provides empirical evidence on the effectiveness of the policy, offering insights for future legislation and suggesting pathways to enhance innovation capacity in the context of ongoing healthcare reform in China.

## 1 Introduction

At present, China’s economy has shifted from rapid growth to a necessary stage of high-quality development. High-quality development is the main theme of today’s economic development. Simultaneously, innovation-driven high-quality development is the key to implementing new development concepts and solving outstanding contradictions and problems in current economic development. Pharmaceutical companies, as benchmark enterprises at the forefront of innovation, recorded a revenue of ¥2,930.4 billion in 2023, representing a year-on-year growth of 7.5%, substantially outpacing the gross domestic product (GDP) growth rate. Pharmaceutical companies have a strong driving force in economic growth and play an obvious leading role. Because of its industrial characteristics of “intensive innovation needs” and “prominent technical needs,” it is even more necessary to serve as a model for the innovation industry, lead the high-quality development of the entire industry, and drive the improvement of the innovation level of the entire industry. However, China’s pharmaceutical industry, as well as the majority of its firms, exhibits the characteristics of being “large in scale but weak in strength” and “numerous in number but lacking competitiveness.” These issues primarily arise from insufficient innovation incentives among pharmaceutical and biotechnology enterprises, a weak propensity for independent innovation, and low efficiency in translating innovation outputs into practical applications, which collectively represent urgent challenges for the entire industry. Current research has largely concentrated on pharmaceutical firms’ internal innovation capabilities and the constraints posed by internal conflicts, while the role of external institutional evolution in driving innovation remains underexplored. Against this backdrop, examining the impact of policies and regulations on pharmaceutical innovation is highly relevant, as it can harness institutional changes to effectively steer and stimulate independent innovation within these firms.

**Figure 1:**
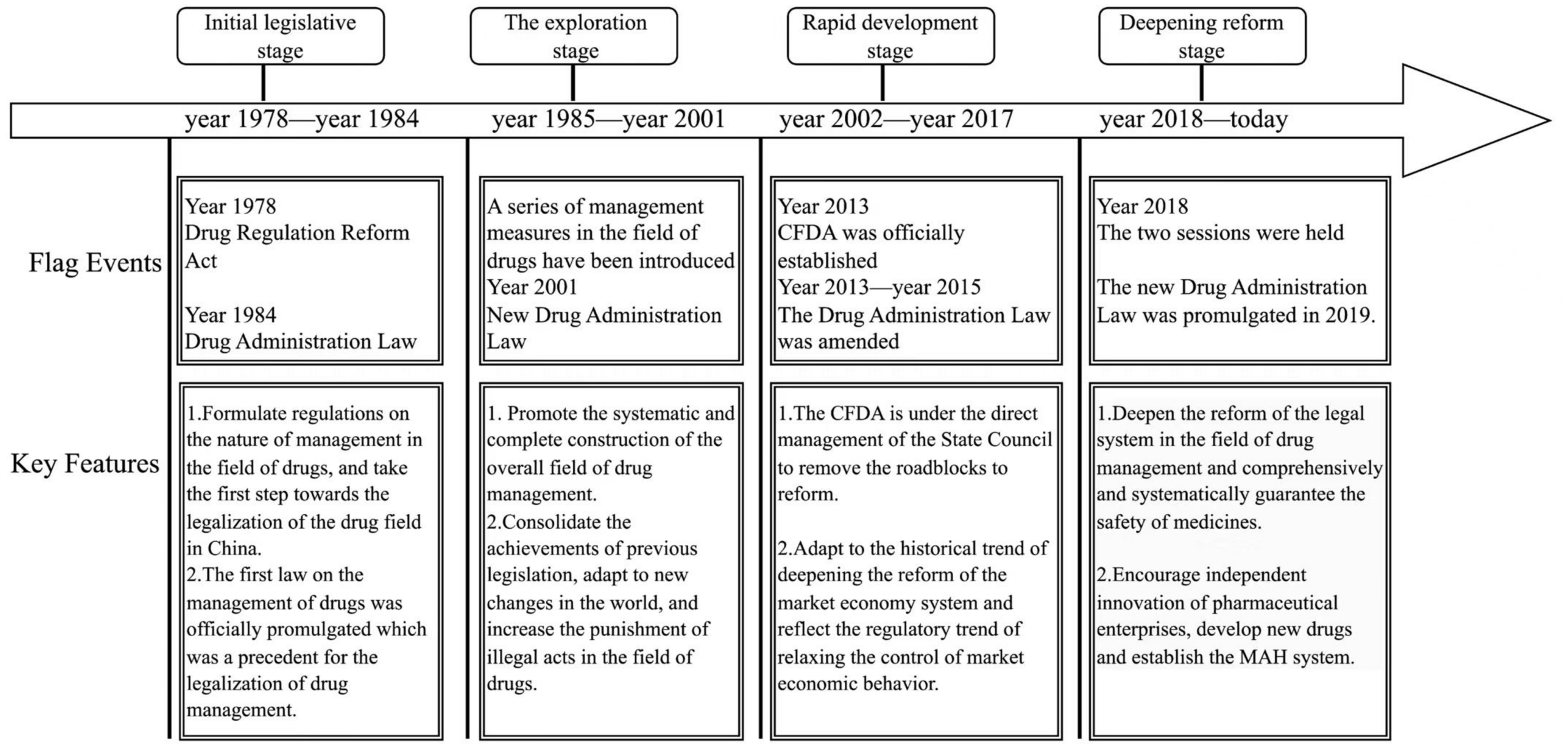
Flow chart of the development process of the drug administration law.

As the fundamental law governing drug administration in China, the Drug Administration Law has continuously evolved in response to changing social needs and regulatory priorities. It has gradually developed from an initial legislative framework into a comprehensive regulatory system, reflecting a shift in regulatory focus from ensuring drug supply to improving drug quality and safety. Against this backdrop, the 2019 revision represents a critical turning point in the legislative evolution of the Drug Administration Law. Compared with previous amendments, this revision introduced systematic and structural reforms within the existing regulatory framework, constituting the second major revision since the promulgation of the Drug Administration Law in 1984. The revised law comprises 12 chapters and 155 articles, covering all key aspects of drug regulation. This study presents the development stages, implementation timeline, key milestones, and main characteristics of China’s Drug Administration Law in a schematic diagram, as illustrated in Figure 1.

The 2019 amendment abolished the former GMP/GSP certification system and established a dedicated chapter to clarify the state’s supportive stance toward marketing authorization holders (MAHs). Furthermore, the revised law strengthened the drug traceability system and pharmacovigilance mechanisms, thereby reinforcing lifecycle-based regulation to ensure medication safety. Notably, with respect to pharmaceutical innovation, the law explicitly emphasizes a clinical value–oriented approach, encourages the development of innovative drugs targeting specific diseases, and introduces priority review and approval pathways. Furthermore, the revised law explicitly incorporates legal protection for the legitimate rights and interests of research entities, including individuals, organizations, and enterprises, which reflects a significant shift in regulatory philosophy toward supporting and incentivizing pharmaceutical innovation, demonstrating the state’s heightened commitment to encouraging innovation within the pharmaceutical industry.

Against this backdrop, several questions remain unresolved: How does the revised Drug Administration Law affect pharmaceutical innovation? Do its impacts vary across different types of firms? What are the underlying mechanisms of its influence? To address these questions, this study used the listed pharmaceutical companies from 2014 to 2023 as the research sample. It leverages the 2019 implementation of the revised Drug Administration Law as a quasi-natural experiment. A sharp regression discontinuity design is employed to examine the law’s impact on pharmaceutical innovation. This approach not only provides a new perspective for understanding the specific effects of the law but also offers empirical evidence to guide policymakers and corporate managers in promoting industry innovation.

The primary contributions of this study are as follows: First, building on existing research, it extends the quantitative analysis of pharmaceutical innovation by empirically examining the impact of the revised Drug Administration Law using a sharp regression discontinuity model, thereby filling a gap in the literature. Second, through mediation analysis, it uncovers the mechanisms of the law’s influence, particularly in terms of financing constraints and human capital investment structure, clarifying its pathways of impact. Third, the study conducts heterogeneity analyses to explore how the law’s effects differ across geographic regions, ownership types, and firm sizes.

## 2 Literature review and theoretical hypotheses

### 2.1 Mechanisms for the impact of the amended drug administration law on innovation in the pharmaceutical industry based on the current situation

Innovation is a key driver of long-term economic growth and productivity. The 2019 revision of the Drug Administration Law emphasizes “supporting innovation” (1) and establishes a comprehensive institutional mechanism to encourage new drug research and development (R&D) (2). It restructures the drug administration system across the full drug lifecycle, reshaping the design logic of the previous law and highlighting the law’s role in promoting scientific and technological innovation in the pharmaceutical industry (3).

Notably, the revision formally endorses the drug marketing authorization holder (MAH) system, which positively stimulates innovation (4). MAHs have legal rights to conduct drug R&D, production, and sales, and can benefit from relevant incentive policies after marketing, thereby enhancing the enthusiasm of research institutions and personnel (5, 6). Furthermore, the law encourages the development of innovative drugs targeting serious or rare diseases, multi-target systemic interventions, and pediatric medicines, while also supporting independent innovation and modernization of traditional Chinese medicines (6–8).

In short, the revision of the “Drug Administration Law” will strictly regulate management and encourage innovation as the main themes throughout the text. It is not only a comprehensive upgrade of the management system of China’s pharmaceutical industry but also a long-term plan for future development, which will certainly promote the industry and provide a strong boost toward realizing the great goal of the strategy of a healthy China.

In summary, this study proposes the first hypothesis:

*H1*: The implementation of the 2019 revision of the “Drug Administration Law” could boost the innovative output of pharmaceutical and biotechnology companies.

### 2.2 Mechanisms of financing constraints affecting innovation in the pharmaceutical industry

Enterprise innovation is highly sensitive to financing constraints. Firms facing severe constraints may forgo valuable projects due to insufficient funds, leading to suboptimal resource allocation (9). Financial constraints significantly reduce participation in innovative activities, particularly in high-tech sectors (10, 11). Externally, banks tend to adopt conservative lending practices (12), and high-tech firms, such as pharmaceutical companies, often lack collateral for financing, exacerbating constraints (13). Internally, these constraints may force firms to abandon positive net present value projects, reduce R&D investment, and lower operational efficiency (14, 15). The revised Drug Administration Law provides explicit signals of governmental support for pharmaceutical innovation, which can alleviate financing constraints, attract external investment, and encourage firms to pursue high-value, quality-driven R&D strategies (16).

From the above analysis, this paper proposes the second hypothesis

*H2*: The 2019 revision of the “Drug Administration Law” can promote corporate innovation output by alleviating their financing constraints and thus promoting their innovation output.

### 2.3 Mechanisms of human capital structure influencing innovation in the pharmaceutical industry

Human capital is a critical strategic resource for sustaining a firm’s competitive advantage due to its value and scarcity (17). As a core driver of innovation, human capital underpins knowledge absorption and creation, enabling firms with high human capital to undertake complex and challenging innovations (18). Empirical evidence indicates that human capital promotes technological innovation (19), and optimizing human capital structure enhances innovation resilience (20). Firms with well-structured human capital can effectively integrate knowledge and apply advanced technologies, improving resource allocation and facilitating disruptive innovation (21). In pursuing frontier technologies, leveraging the quantitative and qualitative advantages of human capital is essential to maximize innovation output efficiency (22). Van Reenen (23) argues that innovation policies are most effective when coupled with measures to expand the supply of STEM talent, strengthen higher education, and lower barriers to entry into innovative occupations. In the context of the pharmaceutical industry, the 2019 reform reduces institutional uncertainty. It stabilizes firms’ expectations, which encourages greater retention of key researchers, while also creating incentives for collaboration with universities and training institutions to broaden the talent pool. Such adjustments help enterprises secure both the quantity and quality of human capital needed for sustained innovation.

The 2019 revision, following this line of reasoning, suggests that the revised law not only reinforces firms’ incentives to increase R&D investment but also drives firms to optimize their human capital structure by increasing the proportion of high-skilled researchers and regulatory experts. Such structural adjustments improve firms’ ability to absorb, integrate, and apply new knowledge, thereby fostering sustained innovative output. This mechanism provides a solid theoretical foundation for the innovation-enhancing effects proposed in this study. The “Drug Administration Law” and its series of supporting policy measures to encourage innovation can prompt enterprises to adjust their human capital structure, thereby improving their innovation capacity.

Therefore, this study proposes the third hypothesis:

*H3*: The 2019 revision of “drug administration law” can promote corporate innovation output by facilitating the restructuring of enterprises' human capital.

## 3 Research design

### 3.1 Sample selection and data sources

The article selects 503 listed companies in the pharmaceutical and biotechnology sectors from 2014 to 2023 for empirical analysis. The sample construction follows these criteria: (1) ST, *ST, and PT stocks are excluded; (2) companies listed for less than 1 year, delisted, or suspended are excluded; and (3) the top and bottom 1% of observations are winsorized to mitigate the influence of outliers on the results. The data are sourced from CSMAR (Guo Tai an Database), and all empirical analyses are conducted using Stata 18.0.

### 3.2 Variable definition

#### 3.2.1 Explained variable: corporate innovation

Researchers (24) showed that the number of design patents is relatively small and the technological content is low. Hence, such patents are not considered. This study used the sum of the number of invention patents and utility model patents of an enterprise to measure the innovation of the enterprise. To enhance the robustness and credibility of the measurement, we further transform the innovation variable by taking ln (Inno + 1).

The amended “Drug Administration Law of the People’s Republic of China” in 2019, as a legal document passed by the National People’s Congress, has had a clear impact on national pharmaceutical and biotechnology enterprises before and after its implementation. The law came into full force on December 1, 2019. Therefore, this study used 2020 as the breakpoint for policy implementation, and empirically analyzes it by using a sharp regression discontinuity design model, with the treatment variables and the driver variables defined as follows:

#### 3.2.2 Treatment variable


$$\mathrm{Di}={\{}_{0\;T_{i}<0}^{1\;T_{i}\geq 0}$$ (1)

As shown in Equation 1, where *D_i_* is a treatment variable indicating whether individual pharmaceutical or biotechnology enterprises are affected by the amended law, with *D = 1* if the enterprise belongs to the experimental group and *D = 0* if it belongs to the control group.

#### 3.2.3 Driver variables (Ti)

As shown in Equation 1, *T_i_* is a driver variable indicating the interval between the time and the year of implementation of the recent Drug Administration Law reform; if *T_i_ ≥ 0*, it means the time is in 2020 or later, and *T_i_ < 0* means before 2020.

#### 3.2.4 Financing constraints (SA)

The article refers to Hadlock andPierce (25) for calculating financing constraints and argues that the larger the absolute value of the SA index, the greater the degree of financing constraints faced by the corporate sector (26). The specific calculation of the SA index is SA = -0.737*Size+0.043*Size^2–0.040*Age, where Size represents the scale of the enterprise, usually in millions of yuan, and Age represents the age of the enterprise. The calculation result of the SA index shows a negative value. The larger its absolute value, the more serious the financing constraints of the enterprise.

#### 3.2.5 Human capital structure (RD)

Referring to the definition of human capital structure by scholars such as Ma and Tian (27), it refers to the proportional composition of the components in the human capital system and their interrelationships. It reflects the proportion of the labor force classified by criteria such as different levels of education and public acceptance among the total labor force. This study defines the ratio of the number of R&D personnel to the total number of employees in an enterprise as the human capital structure according to the personnel structure of the enterprise.

#### 3.2.6 Control variables

Referring to the research by Qiao et al. (28), Liu et al. (29), Qi and Zhu (30) (31), Zhu and Tao (32), this study selected the following variables as control variables: capital structure (Lev), inventory intensity (Inv), board size (Boardsize), the ratio of independent directors (Indep), and dual positions (Cp), TobinQ (TobinQ) are six variables that may have an impact on corporate innovation, and controlling for the above variables can alleviate the problem of omitted-variable bias to a certain extent. The symbols and definitions of the relevant variables are shown in Table 1:

**Table 1:** Variable definition table.

Variable types	Variable name	Variable definition
Explained variable	Corporate Innovation (Inno)	The total number of invention patents and utility model patents is transformed using ln (Inno + 1).
Treatment variables	Implementation of the Newly Revised Drug Administration Law (D)	Are companies affected by the revised Drug Administration Law
Driver variables	Time until the implementation of the revised Drug Administration Law (T)	The gap between the time and the implementation year of the revised Drug Administration Law
Mediating variables	Financing Constraints (SA)	SA index, see above for specific calculation method
	Human capital structure (RD)	Ratio of R&D personnel to total employees in the enterprise
Control variables	Capital Structure (Lev)	The ratio of total liabilities to total assets at the end of the year
	Inventory Intensity (Inv)	Net inventory to total assets ratio
	Board Size (Boardsize)	The natural logarithm of the total number of board members
	Ratio of independent directors (Indep)	The proportion of independent directors to all directors
	Dual Positions (Cp) TobinQ(TobinQ)	If the chairman and general manager are the same person, it is 1; otherwise, it is 0 Ratio of a company’s market value to its total assets

### 3.3 Modeling (time-regression discontinuity design)

Hausman and Rapson (33) first introduced the concept of Regression Discontinuity in Time, RD_iT_, the time-regression discontinuity design takes the time of the policy or program start as the break-point and sets the grouping variables according to the time of the interval, which further analyzes the causal relationship between the driving variables and the explanatory variables and assesses the policy effects in this way (34). The time-regression discontinuity design is primarily used in environmental economics and public organizations (35). In this study, we introduce a time-regression discontinuity design into the field of pharmaceuticals to examine the impact of the 2019 amended law “Drug Administration Law” on the innovation performance of pharmaceutical and biotechnology enterprises. The regression discontinuity design can effectively address endogeneity. Thus, this study did not include individual fixed effects in the econometric model to minimize unnecessary interference with the policy assessment (36). However, during the sample collection period, major events such as the “Coronavirus Disease 2019 (COVID-19) pandemic” and other events or policies may have affected the innovation situation of pharmaceutical and biotechnology enterprises. To exclude the corresponding effects, this study controls for the time effect in the empirical process. The recent Drug Administration Law reform took full effect on December 1, 2019, and the probability of whether the sample is intervened at the breakpoint is certain. Therefore, this study used 2020 as the breakpoint of policy implementation and adopted a sharp regression discontinuity design model for empirical analysis. The mode established in this paper is as follows:


$$\mathrm{INN}O_{\mathrm{it}}=\alpha_{0}+\alpha_{1}D_{\mathrm{it}}+\alpha_{2}T_{\mathrm{it}}+\alpha_{3}{Χ}_{\mathrm{it}}+\Sigma_{\text{YEAR}}+\varepsilon_{\mathrm{it}}$$ (2)

As shown in Equation 2, where *i* and *t* represent the enterprise and year respectively; *INNO_it_* is the explained variable, that is, corporate Innovation; *α_0_* is the constant term; *D_it_* is the treatment variable, *α_1_* which represents the average treatment effect at *t* = 2020; *T_it_* is the driving variable, *α_2_* is the coefficient of the driving variable; *Χ_it_* is a series of covariates defined in the previous article, *α_3_* is their coefficients; *Σ_YEAR_* is the time fixed effect; *ɛ_it_* is the random disturbance term.

Based on the previous theoretical analysis, this study next focuses on the role mechanism of the implementation of the 2019 revision of the “Drug Administration Law” in shaping the impact of innovation output of bio-pharmaceutical enterprises from the aspects of financing constraints and human capital investment. To verify hypotheses 2 and 3, based on the mediated effect stepwise regression method, this study constructs the following model based on model (2) for further analysis.


$$SA_{\mathrm{it}}=\beta_{0}+\beta_{1}D_{\mathrm{it}}+\beta_{2}T_{\mathrm{it}}+\beta_{3}X_{\mathrm{it}}+\Sigma_{\text{YEAR}}+\sigma_{\mathrm{it}}$$ (3)

$$\mathrm{INN}O_{\mathrm{it}}=\gamma_{0}+\gamma_{1}D_{\mathrm{it}}+\gamma_{2}T_{\mathrm{it}}+\gamma_{3}SA_{\mathrm{it}}+\gamma_{4}X_{\mathrm{it}}+\Sigma_{\text{YEAR}}+\nu_{\mathrm{it}}$$ (4)

$$RD_{\mathrm{it}}=\chi_{0}+\chi_{1}D_{\mathrm{it}}+\chi_{2}T_{\mathrm{it}}+\chi_{3}X_{\mathrm{it}}+\Sigma_{\text{YEAR}+}\mathrm{\theta it}$$ (5)

$$\mathrm{INN}O_{\mathrm{it}}=\varphi_{0}+\varphi_{1}D_{\mathrm{it}}+\varphi_{2}T_{\mathrm{it}}+\varphi_{3}RD_{\mathrm{it}}+\varphi_{4}X_{\mathrm{it}}+\Sigma_{\text{YEAR}}+\omega_{\mathrm{it}}$$ (6)

As shown in Equation 3, where *SA_it_* represents financing constraints; as shown in Equation 5, *RD_it_* represents human capital investment; as shown in Equation 4, *INNO_it_* represents enterprise innovation output; *β_0_*, *γ_0_*, *χ_0_*, and *φ_0_* represent constant terms; D*_it_* are treatment variables; *T_it_* are driving variables; *X_it_* are a series of control variables mentioned above; *ΣYEAR* represents time fixed effects; σ_it_, ν_it_, θ_it,_ and ω_it_ are the random error terms. As shown in Equation 4, if the coefficient *γ_2_* is not significant, but *γ_3_* is significant, it means that financing constraints have a complete mediating effect. If both *γ_2_* and *γ_3_* are significant, it means that financing constraints have a partial mediating effect. If *γ_2_* is significant but *γ_3_* is not, this indicates that financing constraints do not have a mediating effect. Similary, As shown in Equation 6, if coefficient *φ_2_* is not significant but *φ_3_* is, this indicates that human capital investment has a complete mediating effect. If both *φ_2_* and *φ_3_* are significant, it indicates that Human capital investment exerts a partial mediating effect. If *φ_2_* is significant but *φ_3_* is not, it means that human capital investment does not have a mediating effect.

## 4 Empirical analysis

### 4.1 Descriptive statistics

First, the variables are 1 and 99% shrinkage processing. The results of descriptive statistics, as shown in Table 2, reflect the general characteristics and distribution of each variable. The maximum value of enterprise innovation (Inno) is 351, and the minimum value is 1, indicating substantial differences in innovation output among different enterprises. The minimum proportion of independent directors is 25%, while the maximum is 60%, suggesting that the proportion of independent directors in each enterprise is relatively reasonable. This reflects the importance of independent directors in corporate governance and helps avoid issues arising from an excessively high proportion of them. The standard deviation of the gearing ratio is 0.175, indicating that enterprises are more consistent in their capital structure management. The standard deviation of board size is 0.181, indicating that the board sizes of enterprises are more similar and that the enterprises may have followed certain industry standard norms. TobinQ varies widely from a minimum of 0.857 to a maximum of 21.296, clearly indicating significant differences among enterprises of different sizes in asset size and market value. The minimum inventory intensity is 0, and the maximum is 0.607, suggesting that enterprises have adopted different operational strategies.

**Table 2:** Descriptive statistics.

Variable	Obs	Mean	Std. dev.	Min	Max
Inno	635	34.847	52.685	1	358
T	635	−0.268	3.013	−6	3
D	635	0.532	0.499	0	1
Indep	635	37.520	5.120	25	60
Cp	635	0.359	0.480	0	1
Lev	635	0.285	0.175	0.025	1.223
Boardsize	635	2.126	0.180	1.609	2.708
TobinQ	635	2.698	1.742	0.857	21.296
Inv	635	0.091	0.063	0	0.607

### 4.2 OLS regression

Before regression, this study conducted the F-test and the Hausman test. The results showed that it is more appropriate to choose the fixed-effects model for regression (limited to space, the process is omitted). This study is initially based on ordinary least squares regression analysis of the sample enterprises to compare with the results of the subsequent regression of the discontinuity. The results are shown in Table 3, where column (1) presents the basic regression, with a treatment effect coefficient of 2.804, significant at the 1% level. In column (2), based on (1), adding covariates reduces the coefficient to 2.702, still significant at the 1% level. In column (3), based on (2), adding covariates reduces the coefficient to 2.702, still significant at the 1% level. In column (3), controls for the time effect based on (2), the coefficient decreases to 2.695, but is still significant at the 1% level, indicating that the implementation of the amended law “Drug Administration Law” provides a favorable institutional environment for the innovation of pharmaceutical and biotechnology enterprises. This result preliminarily verifies hypothesis 1 of this study, that the implementation of the amended law “Drug Administration Law” can promote the pharmaceutical and biotechnology enterprises’ innovation output.

**Table 3:** OLS regression.

Group	(1)	(2)	(3)
Treatment effect	2.804 ^***^ (5.54)	2.702 ^***^ (5.22)	2.695 ^***^ (3.96)
Control variables	No	Yes	Yes
Time fixed effects	No	No	Yes
Number of samples	635	635	635
*R* ^2^	0.0368	0.0415	0.0501

*, **, *** indicate significance at the 10, 5, and 1% levels, respectively, with standard errors in parentheses.

### 4.3 Applicability test for regression discontinuity

#### 4.3.1 Driving variables are not subject to human control tests

One prerequisite for applying the regression discontinuity design is that the driving variable must be exogenous. This assumption can be tested for manipulation by examining the uniformity of the sample distribution in the vicinity of the breakpoint through methods such as the McCrary density test (37). Since the driving variable is time in the regression discontinuity design framework and is unaffected by the implementation of the amended law, the Drag Administration Law depends solely on the year in which the observed sample enterprises are in and cannot be determined by the subjective choice of the enterprises (35). Therefore, the research sample selected in this study will not be manipulated around the policy breakpoint, and the validity of the coefficient estimates can be guaranteed.

#### 4.3.2 Covariate smoothness test

**Figure 2:**
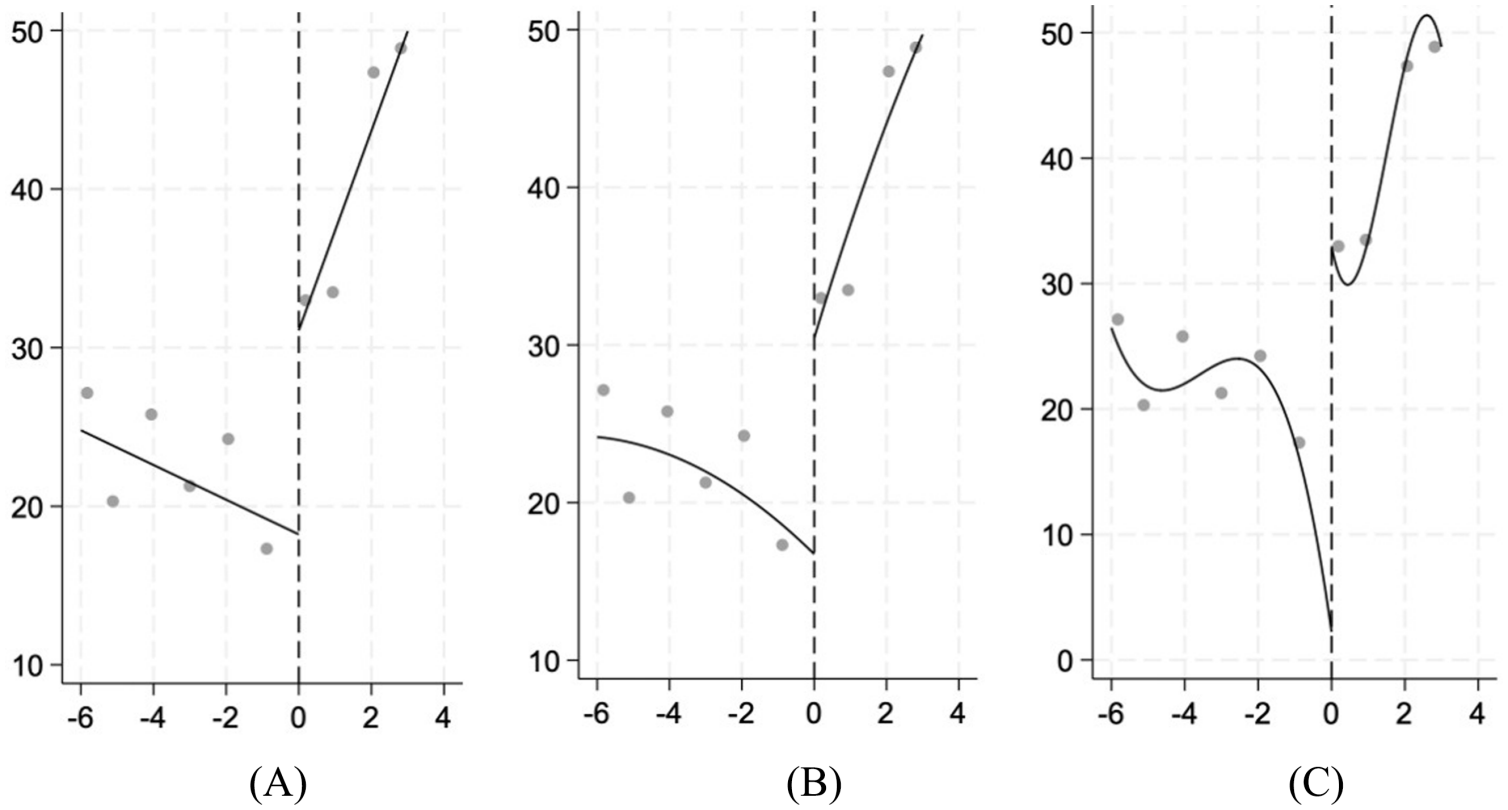
Discontinuity effect test. linear **(A)**, second-order **(B)**, and third-order **(C)**.

Testing the continuity of the covariates before and after the breakpoints is essential; the validity of the regression discontinuity design relies on the continuity assumption, which requires the covariates in the regression to be smooth at the breakpoints (38). In this study, the covariates are used as explanatory variables, and regression analysis is conducted under their respective optimal bandwidths to test the continuity of the covariates at the breakpoint (39). The test results are shown in Table 4, where none of the covariates pass the significance test, indicating that all the control variables are continuous. This suggests that the breakpoints observed in the empirical results are solely due to the implementation of the amended law “Drug Administration Law,” and are unrelated to the other characteristic variables. The design of the discontinuity in this study complies with the assumption on the covariate smoothness, which allows for the next step in the analysis (Figure 2).

**Table 4:** Covariate smoothness test.

Variable	(1)	(2)	(3)	(4)	(5)	(6)
	Cp	Indep	Lev	Boardsize	TobinQ	Inv
Treatment Effect	−0.005 (−0.84)	0.037 (0.61)	0.002 (0.82)	−0.002 (−0.96)	−0.038 (−1.33)	−0.001 (−0.35)
Control	Yes	Yes	Yes	Yes	Yes	Yes
Time fixed effects	Yes	Yes	Yes	Yes	Yes	Yes
Sample size	635	635	635	635	635	635

#### 4.3.3 Discontinuity effect test

Before the empirical analysis, it is also necessary to test whether corporate innovation has changed significantly on either side of the breakpoint (41). Figure 2 presents the results of linear (A), second-order (B), and third-order (C) fits of corporate innovation to the driving variables, with the horizontal axis showing the difference between the year in which the sample enterprises are located and 2020. The figure clearly shows a clear upward jump in corporate innovation at the zero point, indicating that the innovation of pharmaceutical and biotechnology enterprises has risen considerably after the promulgation and implementation of the amended law “Drug Administration Law,” which can be further tested to verify the specific implementation effect of the 2019 revision “Drug Administration Law”.

#### 4.3.4 Benchmark regression

In the context of regression discontinuity design, the current academic community has not yet established a generally recognized standard for selecting parametric and non-parametric estimation methods. Based on previous research findings from scholars both domestically and internationally, parametric estimation methods often face sensitivity issues in selecting the polynomial order, which increases the uncertainty of the results. Simultaneously, parametric estimation also has limitations, including inefficient computation, large errors, and insufficient accuracy. Conversely, non-parametric estimation methods offer greater flexibility, as they do not rely on a specific functional form and can directly analyze complex relationships between variables, thus potentially providing more accurate and in-depth insights (40). Therefore, non-parametric estimation is chosen as the baseline regression method in this paper. The non-parametric regression results of optimal bandwidth and triangular kernel function are given in column (1) of Table 5; column (2) adds covariates based on (1) to carry out the above regression, and the results show that the estimated coefficients before and after the addition of covariates are 15.611 and 16.893, respectively, which are both significant at the 5% level, indicating that the 2019 revision “Drug Administration Law” has an obvious promotional effect on the innovation output of enterprises, and hypothesis l is verified.

**Table 5:** Nonparametric estimation results.

Group	(1)	(2)
Treatment effect	15.611** (2.16)	16.893** (2.32)
Kernel	Triangular	Triangular
Bandwidth	4.018	4.018
Control	No	Yes
Number of samples	635	635

*, **, *** indicate significance at the 10, 5, and 1% levels, respectively, with standard errors in parentheses.

## 5 Analysis of mechanisms of action

In this study, financing constraints and human capital investment of enterprises were used as dependent variables for regression estimation. Table 6 reports the results obtained with financing constraints as mediating variables, columns (1) and (2) are the basic regression results, and the coefficient of column (1) is −0.0408, which is significant at 1% level, indicating that the implementation of the 2019 revision “Drug Administration Law” can inhibit the financing constraints of enterprises. Constraint has a coefficient of 2.00, which is significant at the 5% level; that is, the implementation of the 2019 revision of the “Drug Administration Law” can promote corporate innovation output by suppressing corporate financing constraints, and hypothesis 2 is verified. Columns (3) and (4) control for the time fixed effect based on columns (1) and (2), and the coefficient’s value is changed, but the level of significance remains unchanged. Columns (5) and (6) have the same coefficient based on columns (3) and (4). Although the coefficients have declined, they remain significant at the 1 and 5% level, indicating the reliability and robustness of the above conclusion and further validating hypothesis 2. With the implementation of the 2019 revision of the “Drug Administration Law,” the overall environment of the pharmaceutical industry has improved, and the R&D capabilities and market competitiveness of enterprises have been enhanced, which may enable enterprises to have more opportunities and channels for financing, such as bank loans, venture capital, and listed financing. Furthermore, the 2019 revision of the “Drug Administration Law” has more stringent regulations on drug R&D, production, and quality management. The enterprises’ own credibility has been significantly enhanced, which may somewhat ease financial difficulties and reduce costs, making investors more confident in assessing financing risk for the enterprises. As a result, they will be willing to provide them with more financial support.

**Table 6:** Mediated effects regressions for SA.

Variable	(1)	(2)	(3)	(4)	(5)	(6)
	SA	Inno	SA	Inno	SA	Inno
*t*	−0.0408^***^ (−61.76)	0.0971^***^ (6.67)	−0.0409^***^ (−50.00)	0.0994^***^ (5.47)	−0.041^***^ (−43.95)	0.1004^***^ (5.45)
SA		0.5531^**^ (2.00)		0.5174** (1.89)		0.5598** (1.93)
control	No	No	No	No	Yes	Yes
Time fixed effects	No	No	Yes	Yes	Yes	Yes
*N*	635	635	635	635	635	635

*, **, *** indicate significance at the 10, 5, and 1% levels, respectively, with standard errors in parentheses.

Table 7 reports the results obtained from human capital investment as a mediating variable. Columns (1) and (2) show the results of the benchmark regression. The coefficient reported in column (1) is 0.491 and is significant at 1% level, which indicates that the implementation of the 2019 revision “Drug Administration Law” can significantly promote the investment of human capital in enterprises. The results in column (2) indicate that both the implementation of the 2019 revision of the “Drug Administration Law” and the investment in human capital and innovation outputs of enterprises are positively related, implying that both ***φ***_***2***_ and ***φ***_***3***_ model (6) are significant. Furthermore, human capital investment has a partial mediating effect on the implementation of the 2019 revision of the Drug Administration Law in promoting the innovation output of the enterprise through increased investment in human capital, verifying hypothesis 3. Columns (3) and (4) are the control for the time fixed effect based on columns (1) and (2). Columns (5) and (6) further add covariates based on (3) and (4). Although the coefficients have changed, all coefficients remain significant at the 1% level, indicating the robustness of the above findings and further verifying hypothesis 3. High-quality human capital is the core of innovative activities of enterprises, and in the pharmaceutical industry, high-level pharmaceutical technology is the key to the innovative development of enterprises. Research and development personnel are the backbone of this process; therefore, having high-quality, highly skilled R&D personnel is key to the innovative activities of pharmaceutical enterprises. The implementation of the updated “Drug Administration Law” can boost innovation outputs of enterprises, such as human capital investment, by increasing these investments through the enforcement of the new revised “Drug Administration Law.” The implementation of the updated Drug Administration Law can further enhance innovation capacity by providing a more relaxed and efficient legal environment for corporate innovation, promoting increases in innovation inputs such as human capital investment of enterprises, and thus promoting the healthy development of the pharmaceutical industry.

**Table 7:** Mediated effects regression for RD.

Variable	(1)	(2)	(3)	(4)	(5)	(6)
	RD	Inno	RD	Inno	RD	Inno
*t*	0.491*** (5.66)	0.0709^***^ (5.27)	0.437*** (4.47)	0.0598^***^ (4.01)	0.508*** (4.86)	0.0457^***^ (2.87)
RD		0.0138^***^ (3.20)		0.0133*** (3.10)		0.142*** (3.22)
control	No	No	No	No	Yes	Yes
Time fixed effects	No	No	Yes	Yes	Yes	Yes
*N*	599	599	599	599	599	599

*, **, *** indicate significance at the 10, 5, and 1% levels, respectively, with standard errors in parentheses.

## 6 Further analysis

### 6.1 Business location

To compare the extent to which different regions of China are affected by the implementation of the revised drug management, this study is divided into three sub-samples based on the regions where the enterprises are located: eastern, central, and western regions. The specific division of the 31 provinces, municipalities, and autonomous regions is shown in Table 8.

**Table 8:** Eastern central western division.

Area	Provinces, municipalities, and autonomous regions included
Eastern Region	Beijing, Tianjin, Hebei, Shanghai, Jiangsu, Zhejiang, Fujian, Shandong, Guangdong, Guangxi, Hainan, Liaoning
Central Region	Jilin Province, Heilongjiang Province, Shanxi Province, Anhui Province, Jiangxi Province, Henan Province, Hubei Province, Hunan Province, Inner Mongolia Autonomous Region
Western Region	Chongqing, Sichuan, Guizhou, Yunnan, Tibet, Shaanxi, Gansu, Ningxia, Qinghai, Xinjiang

The impact of the 2019 revision of the Drug Administration Law implementation on different regions of China is shown in Table 9. The effect of the implementation of the new “Drug Administration Law” on innovation output in the eastern region is significant at the 5 and 1% levels before and after the addition of the covariates, whereas the implementation of the new revised “Drug Administration Law” has a negative and non-significant effect on the innovation output of enterprises in the central and western regions. The eastern region has a stronger economic foundation, which can provide sufficient financial support for pharmaceutical innovation, whether through R&D investment, equipment purchases, or talent introduction. Simultaneously, the eastern region tends to have more intensive scientific research resources, with universities, research institutions, and enterprises engaging in closer cooperation among industry, academia, and research, and can quickly transform scientific research results into actual innovation output. Moreover, the market environment in the eastern region is more open and mature, with greater acceptance of innovation outcomes, which motivates enterprises to invest more actively in innovation and to realize its value more quickly. Conversely, the central and western regions may lack the capacity to support large-scale R&D due to their relatively weak economic strength. Furthermore, the relative lack of research resources in the central and western regions makes it difficult for enterprises to access cutting-edge technologies and ideas, leading to inefficient innovation. The market activity in the central and western regions is also insufficient, which may limit the market transformation of innovations and make it difficult for enterprises to quickly realize the commercial value of their innovations, even if they have some. As a result, it fails to show a significant increase in innovation output or may even have a negative effect after the implementation of the updated “Drug Administration Law.”

**Table 9:** Heterogeneity test of the region where the enterprises are located.

Variable	(1)		(2)		(3)	
	Eastern region		Central region		Western region	
Treatment effect	14.076** (1.96)	21.274*** (2.92)	11.216 (−0.82)	−1.886 (−0.11)	1.733 (−0.35)	1.242 (−0.12)
Kernel	Triangular		Triangular		Triangular	
Control	No	Yes	No	Yes	No	Yes
Bandwidth	4.176		4.455		1.818	
*N*	473		91		71	

*, **, *** indicate significance at the 10, 5, and 1% levels, respectively, with standard errors in parentheses.

### 6.2 Nature of property rights

In order to test the effect of the implementation of the 2019 revision of the Drug Administration Law on enterprises of different natures, this study further distinguishes the sample enterprises into state-owned enterprises and non-state-owned enterprises for heterogeneity analysis, and the results obtained are shown in Table 10. The results in column (1) show that the treatment effects of the implementation of the 2019 revision of the Drug Administration Law on state-owned enterprises before and after the addition of the covariates are 27.990 and 30.655, which are significant at the 10 and 5% levels, respectively. This suggests that the implementation of the 2019 revision of the Drug Administration Law significantly promotes the innovation output of state-owned biomedical enterprises. Conversely, the results in column (2) show that the implementation of the 2019 revision of the Drug Administration Law on non-state-owned enterprises has a significant impact on innovation output. The implementation of the Administration Law has the same positive but insignificant effect on non-state-owned enterprises. Compared to non-state-owned enterprises, state-owned enterprises (SOEs) tend to have a stronger sense of compliance and can quickly adapt to changes in regulations after the implementation of the 2019 revision of the Drug Administration Law, ensuring their operations comply with the law. Furthermore, SOEs have a closer relationship with the government and may be more likely to receive policy guidance and support, while non-SOEs may face more uncertainty and challenges.

**Table 10:** Heterogeneity test of enterprise property rights.

Variable	(1)		(2)	
	State-owned		Nonstate owned	
Treatment effect	27.990* (1.81)	30.655** (2.14)	12.178 (1.38)	13.163 (1.46)
Kernel	Triangular		Triangular	
Control	No	Yes	No	Yes
Bandwidth	3.168		3.885	
*N*	97		519	

*, **, *** indicate significance at the 10, 5, and 1% levels, respectively, with standard errors in parentheses.

### 6.3 Enterprise scale

Under the implementation of the updated “Drug Administration Law,” the size of the enterprise may influence its innovation output (42). This study divides the sample enterprises into large, medium, and small enterprises based on the scale of the total assets. Specifically, enterprises with total assets of at least 10 billion are large-scale, those with less than 1 billion are small-scale, and the rest are medium-sized. The results of heterogeneity analysis by enterprise size are shown in Table 11. As illustrated in Table 12, the impact of the implementation of the 2019 Drug Administration Law on medium-sized enterprises before and after adding covariates is 13.082 and 12.707, respectively, both of which are significantly positive at the 10% level for large- and small-scale enterprises. The effect of enterprise size is also positive but not significant. This might be because medium-sized enterprises are relatively flexible in resource allocation and can adjust their business direction following the government’s footsteps, thereby achieving rapid development. Large-scale enterprises usually have sufficient resources. However, due to the large scale of the enterprise, it is relatively difficult to make timely adjustments in response to the requirements of the updated “Drug Administration Law.” Therefore, although large-scale enterprises will also actively respond to legal requirements, the impact may not be as significant as that of medium-sized enterprises. For small-scale enterprises, resources are relatively limited, making it difficult to meet certain requirements of the 2019 Drug Administration Law within a short period of time. This could lead the enterprise to face more challenges and, if it cannot comply with legal requirements, to termination if it is forced to withdraw from the market.

**Table 11:** Heterogeneity test for enterprise size.

Variable	(1)		(2)		(3)	
	Large		Medium		Small	
Treatment effect	28.738 (1.60)	26.044 (1.33)	13.082* (1.85)	12.707* (1.78)	10.644 (1.45)	6.793 (0.82)
Kernel	Triangular		Triangular		Triangular	
Control	No	Yes	No	Yes	No	Yes
Bandwidth	2.663		4.644		3.553	
*N*	98		487		50	

*, **, *** indicate significance at the 10, 5, and 1% levels, respectively, with standard errors in parentheses.

**Table 12:** Placebo test.

Variable	(1)		(2)		(3)		(4)	
	2018		2019		2021		2022	
Treatment effect	−4.535 (−0.39)	−7.506 (−0.63)	−5.245 (−0.80)	−5.026 (−0.78)	−14.127 (−1.19)	15.900 (−1.36)	8.143 (0.91)	9.399 (0.98)
Kernel	Triangular		Triangular		Triangular		Triangular	
Control	No	Yes	No	Yes	No	Yes	No	Yes
Bandwidth	5.772		5.187		2.818		4.186	
*N*	635		635		635		635	

*, **, *** indicate significance at the 10, 5, and 1% levels, respectively, with standard errors in parentheses.

## 7 Robustness tests

### 7.1 Placebo test

Changes in enterprise innovation around 2020 may be caused by other random factors. Therefore, different policy years are further selected as explanatory variables for placebo tests, and linear regression is performed with 2018, 2019, 2021, and 2022 as breakpoints. If the treatment effect is significant at the pseudo-breakpoint, this indicates that the sharp regression discontinuity design is invalid. Otherwise, it suggests that the benchmark regression results are robust. As shown in Table 12, when the breakpoints are changed to other years, the treatment effects are not significant, indicating that the conclusions drawn in this article are robust and reliable.

### 7.2 Bandwidth dependence test

Selecting different bandwidths will affect the accuracy of the estimation results. Table 13 shows the estimation results for selecting 0.50, 0.75, 1.25, 1.50, and 1.75 times the optimal bandwidth using the triangular kernel function. As shown in Table 13, although changing the bandwidth causes fluctuations in the regression results, all results remain significant across different bandwidths, indicating that the estimation results of this article are robust.

**Table 13:** Bandwidth dependence tests.

Group	(1)	(2)	(3)	(4)	(5)
Bandwidth	0.50	0.75	1.25	1.50	1.75
Treatment effect	22.455*** (2.67)	19.762** (2.52)	15.125** (2.46)	13.249** (2.43)	13.071** (2.39)
Kernel	Triangular	Triangular	Triangular	Triangular	Triangular
*N*	635	635	635	635	635

*, **, *** indicate significance at the 10, 5, and 1% levels, respectively, with standard errors in parentheses.

### 7.3 Test of kernel function and polynomial order

Table 14 presents the results of nonparametric estimation using the mserd method to select bandwidth. Column (1) shows the estimation result under the first-order polynomial of the trigonometric kernel function. The treatment effect is 32.957, which is significant at the 1% level. Column (2) is replaced with a second-order polynomial based on Column (1), and the coefficient decreases, but it remains significant at the 10% level. Column (3) is estimated based on Column (1) by replacing it with a rectangular kernel function. Column (4) presents the estimates based on Column (2) by replacing it with a rectangular kernel. The results do not change substantially, which demonstrates the reliability of the conclusions of this article.

**Table 14:** Tests of kernel functions and polynomial orders.

Group	(1)	(2)	(3)	(4)
Kernel	Triangular	Triangular	Epanechnikov	Epanechnikov
Polynomial order	1	2	1	2
Treatment effect	32.957*** (6.115)	31.140* (1.839)	32.957*** (6.115)	30.679* (1.811)
*N*	635	635	635	635

*, **, *** indicate significance at the 10, 5, and 1% levels, respectively, with standard errors in parentheses.

### 7.4 Change the way of measuring the explained variable

Replace the original explained variable with the number of invention patents applied for by the enterprise that year, and estimate it using the triangular kernel and optimal bandwidth. The regression results are shown in Table 15. The treatment effects before and after controlling for covariates are significant at the 5% level, further confirming the robustness and reliability of the previous results and once again supporting hypothesis 1. Hence, the implementation of the 2019 Drug Administration Law can promote the output of corporate innovation activities.

**Table 15:** Regression results with replacement of the explained variable.

Group	(1)	(2)
Treatment effect	11.313** (2.14)	12.235** (2.40)
Kernel	Triangular	Triangular
Control	No	Yes
Bandwidth	3.158	
*N*	631	

*, **, *** indicate significance at the 10, 5, and 1% levels, respectively, with standard errors in parentheses.

## 8 Conclusions and recommendations

### 8.1 Discussion

Considering the 2019 revised Drug Administration Law as a quasi-natural experiment, this study employed panel data of listed pharmaceutical enterprises from 2014 to 2023 and applied a regression discontinuity design to systematically examine the policy’s impact on corporate innovation output. The results presented are as follows: First, the revised Drug Administration Law significantly promotes innovation output among pharmaceutical firms, indicating that regulatory strengthening can simultaneously generate effective innovation incentives. Second, mechanism analysis reveals that the policy stimulates innovation primarily by alleviating firms’ financing constraints and optimizing the structure of human capital investment. Third, the heterogeneity analysis demonstrates that policy effects vary across firms: the innovation-promoting effect is more pronounced for enterprises located in eastern regions, state-owned enterprises, and medium-sized firms, whereas the responses of central and western enterprises, non-state-owned enterprises, and small-scale firms are relatively limited. Overall, while enhancing industry standardization, the revised Drug Administration Law exerts a structural influence on firms’ innovation behavior (43).

### 8.2 Policy implications

To further strengthen the innovation-driven effects of institutional reform, this study proposed policy recommendations from both governmental and corporate perspectives.

At the governmental level, it is necessary to consolidate legislative achievements, reinforce policy implementation and dynamic supervision, and enhance the stability and authority of legal enforcement (44). The government should improve the balance of incentives and constraints, accelerate the development of supporting regulations, and establish a coordinated regulatory framework covering the entire life cycle of pharmaceuticals. Moreover, optimizing the allocation of policy resources—particularly by increasing support for central and western regions as well as small and medium-sized enterprises—can help mitigate regional and scale disparities in innovation and promote the coordinated development of the pharmaceutical industry.

At the enterprise level, firms should strengthen their institutional adaptability and strategic responsiveness. Enterprises in eastern regions may further enhance original R&D investment based on existing resource advantages, while firms in central and western regions should expand industry–university–research collaboration and technology acquisition to improve the commercialization efficiency of innovation. State-owned enterprises need to enhance the efficiency of resource allocation, whereas non-state-owned enterprises should improve corporate governance and diversify financing channels. Firms of different sizes should formulate innovation strategies aligned with their resource endowments and organizational flexibility, thereby achieving the coordinated development of regulatory compliance and sustained innovation under evolving institutional environments.

### 8.3 Limitations and future research

Although this study treats the implementation of the 2019 revised Drug Administration Law as a quasi-natural experiment and applies a regression discontinuity design to identify the impact of legislation on corporate innovation—representing a relatively novel methodological approach—several limitations remain. The pharmaceutical industry is structurally complex, and firms differ substantially in size, technological orientation, corporate culture, and innovation strategies. For example, some firms primarily focus on the production of low-cost generic drugs, with innovation centered on process improvement and cost optimization, whereas others specialize in cutting-edge biotechnology, which carries higher risks but potentially higher returns. Such structural differences may lead to heterogeneous responses to legislative shocks, which this study cannot fully capture.

Furthermore, innovation performance is measured mainly through quantifiable output indicators, which may not adequately reflect innovation quality or long-term technological accumulation. Moreover, policy effects may exhibit dynamic and lagged characteristics, and their long-term impact requires further examination. Future research could incorporate firm-level technological heterogeneity, introduce multidimensional indicators of innovation quality and value, and extend the observation period to provide a more comprehensive evaluation of the long-term effects of government legislation on pharmaceutical innovation (45).

## Data Availability

Publicly available datasets were analyzed in this study. This data can be found at: http://www.csmar.com/.
